# Development and validation of a machine learning-based predictive model for prognosis in cerebral hemorrhage patients after hyperbaric oxygen therapy

**DOI:** 10.4103/mgr.MEDGASRES-D-25-00168

**Published:** 2026-09-12

**Authors:** Congyi Ding, Jun Wu, Guangning Zhang, Xiaoming Feng, Jieyang Zhu

**Affiliations:** 1 Department of Neurosurgery Surgery, The First Hospital of Jiaxing, Jiaxing, Zhejiang Province, China; 2 Department of Orthopedic, The First Hospital of Jiaxing, Jiaxing, Zhejiang Province, China

**Keywords:** decision curve analysis, Glasgow coma scale score, hyperbaric oxygen therapy, intracerebral hemorrhage, machine learning, modified Rankin scale, nomogram, prognosis, random forest model, SHapley Additive exPlanations

## Abstract

Intracerebral hemorrhage is a common and critical neurological emergency, and the inconsistent results of hyperbaric oxygen therapy make predicting outcomes challenging. Therefore, a reliable predictive model for outcomes is needed to improve treatment plans. This study used interpretable machine learning to determine key risk factors linked to adverse outcomes in cerebral hemorrhage patient post-hyperbaric oxygen therapy. To improve prognostic accuracy and support clinical decision-making, we analyzed the data from 401 patients with intracerebral hemorrhage who received hyperbaric oxygen therapy at the First Affiliated Hospital of Jiaxing, China, using multivariate logistic regression and machine learning to develop predictive models, with model performance assessed via 10-fold cross-validation. SHapley Additive exPlanations values aided in model interpretation and in the development of a logistic regression-based nomogram. Based on the modified Rankin scale, patients were stratified into two prognostic groups: a good prognosis group (modified Rankin scale < 3, *n* = 265) and a poor prognosis group (modified Rankin scale ≥ 3, *n* = 136). Significant differences in various factors were observed between the groups. Independent risk factors identified included the Glasgow coma scale score, computed tomography hemorrhage volume, activities of daily living score, treatment modality, intraventricular bleeding, and rebleeding. Among the models tested, the random forest algorithm yielded the highest predictive performance (the area under the receiver operating characteristic curve = 0.99). The study identified key predictors, including activities of daily living score, computed tomography hemorrhage volume, and Glasgow coma scale score, as factors influencing hyperbaric oxygen therapy outcomes in intracerebral hemorrhage patients. Random Forest model demonstrated strong predictive capability, with SHapley Additive exPlanations analysis providing insights into individual risk factors. Overall, these findings are crucial for improving clinical decision-making and personalized treatment.

## Graphical abstract

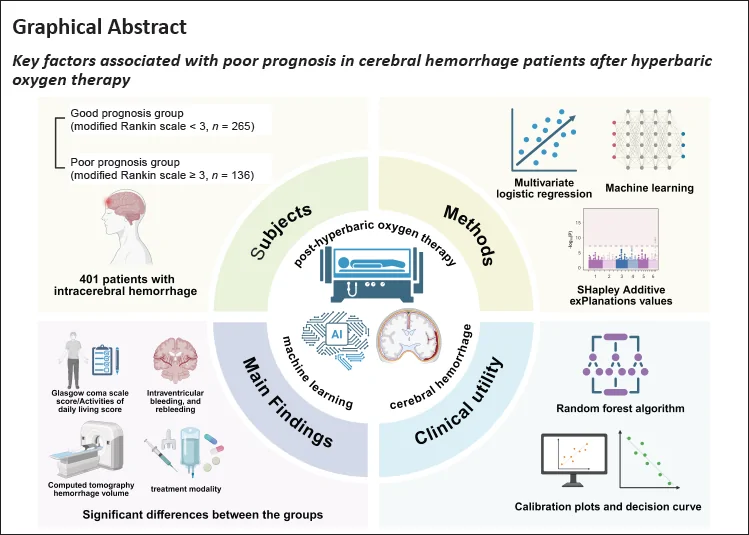

## Introduction

Intracerebral hemorrhage is a severe cerebrovascular event with high mortality rates, comprising 10–15% of all strokes.^1^ Current evidence suggests that the hematoma, cerebral edema, and neuroinflammatory after intracerebral hemorrhage often result in severe neurological deficits and death.^2^,^3^ Surgical craniotomy, catheter drainage, and conservative management have demonstrated limited efficacy in the survival and prognosis of intracerebral hemorrhage patients. Therefore, there is an urgent need to develop more effective treatment strategies.

Hyperbaric oxygen therapy (HBOT) is a promising non-invasive treatment for cerebral hemorrhage recovery. It enhances the oxygenation of brain tissue by increasing the partial pressure of oxygen in ischemic areas, thereby improving dissolved oxygen levels and extending the oxygen diffusion radius. This process helps reduce brain swelling and promotes neural function recovery.^4^ HBOT also reduces ischemia-reperfusion injury^5^ and inflammation, aiding neural repair. Efficacy depends on treatment timing and the patient’s clinical status.^6^,^7^ Identifying ideal candidates and determining prognostic factors are essential for optimizing clinical use of HBOT.

Advancements in big data and computing have expanded the application of machine learning in medicine. Machine learning techniques can analyze complex clinical data to identify prognostic markers and create predictive models.^8^ Techniques such as SHapley Additive exPlanations (SHAP) can explain the contributions of factors to model predictions,^9^ aiding clinicians in treatment optimization. This study aimed to develop an interpretable prognostic model for HBOT, by integrating predictive analytics with explainable artificial intelligence, with the goal of personalizing treatment regimens, reducing inefficiencies, and enhancing patient outcomes, ultimately providing valuable clinical insights in intracerebral hemorrhage patients.

## Methods

### Subjects

A retrospective screening was conducted on 500 patients who received HBOT following cerebral hemorrhage at Jiaxing First Hospital from January 2022 to February 2025. The inclusion criteria comprised: (1) Diagnosis of initial primary or traumatic cerebral hemorrhage via head computed tomography (CT) or magnetic resonance imaging, irrespective of bleeding location (supratentorial/infratentorial); (2) Age between 18 and 80 years with complete baseline clinical data; (3) Completion of at least 3 months of follow-up before discharge or study conclusion. Exclusion criteria included: (1) Contraindications to HBOT; (2) Severe cardiopulmonary dysfunction, hepatorenal insufficiency, active malignant tumors, or hematological disorders.

Following the application of the inclusion and exclusion criteria, 401 patients who received HBOT post-cerebral hemorrhage were included in the study. **Figure 1** shows the flowchart of this study. Neurological recovery and disability were assessed using the modified Rankin scale (mRS).^10^ Patients were categorized into two groups: a good prognosis group (mRS < 3; *n* = 265) and a poor prognosis group (mRS ≥ 3; *n* = 136). The study was approved by the Ethics Committee of Jiaxing First Hospital, Affiliated Hospital of Jiaxing University (approval No. 2025-LP-245; on March 21, 2025) with waivering informed consent. In addition, this study was performed in line with the international ethical guidelines for studies involving human subjects according to the Declaration of Helsinki.

**Figure 1:**
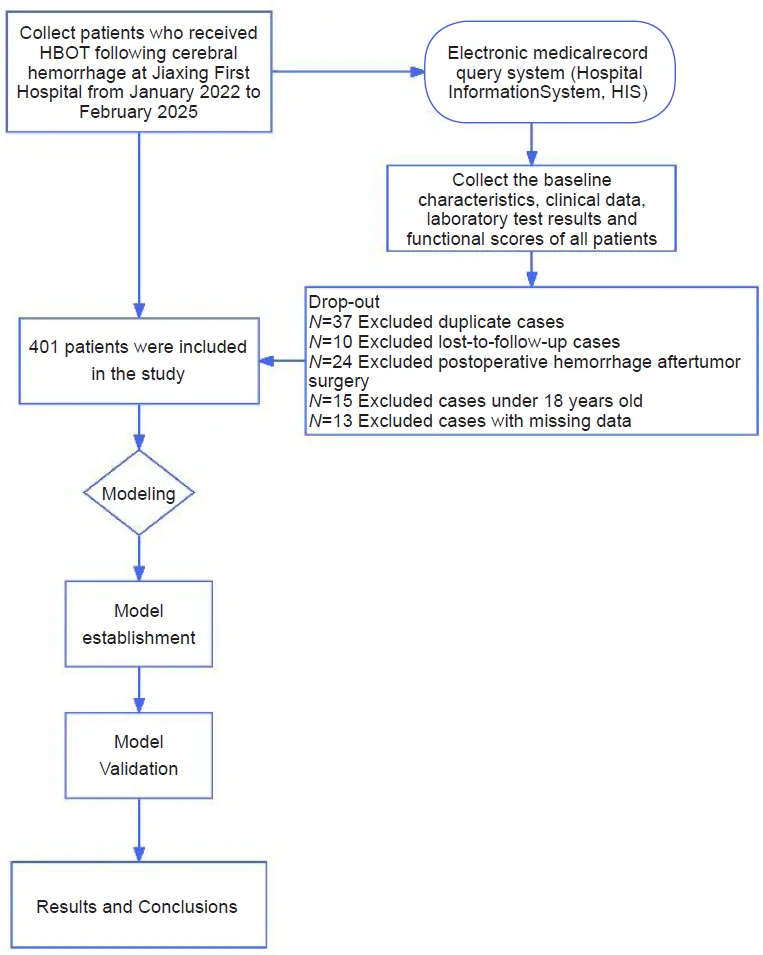
A flowchart of this study This flowchart details patient selection, exclusion criteria, and the subsequent modeling and validation process for the analyzed cohort (*n* = 401). HBOT: Hyperbaric oxygen therapy.

### Data collection

We collected comprehensive patient data from the Hospital Information System, including demographic characteristics (age, gender), medical history (hypertension, diabetes, anticoagulant use), etiology of cerebral hemorrhage (spontaneous, traumatic), treatment modalities (conservative, surgical), surgical approaches (craniotomy, minimally invasive), surgical details (operation time, intraoperative blood loss), admission status (Glasgow coma scale (GCS), activities of daily living (ADL), hemiplegia, aphasia), neuroimaging findings (hemorrhage location, volume), bleeding sites (basal ganglia, thalamus, cerebral lobe, brainstem, cerebellum, subarachnoid hemorrhage, subdural space, epidural space, intravascular hemolysis (IVH)), laboratory values (white blood cell count, hemoglobin count, platelet count, prothrombin time, international normalized ratio, activated partial thromboplastin time, D-dimer, alanine transaminase, aspartate transaminase, albumin, potassium concentration, sodium concentration, calcium concentration, serum creatinine, uric acid, blood glucose), HBOT (timing, frequency), reasons for treatment interruption, HBOT-related complications (oxygen toxicity, barotrauma), other clinical complications (rebleeding, infection, thrombosis, epilepsy), and long-term outcomes (ADL at discharge, intensive care unit/hospital length of stay, 3-month mRS).

### Statistical analysis

All statistical analyses were conducted using SPSS version 25.0 (IBM Corp., Armonk, NY, USA). Normally distributed measurement data are reported as mean ± standard deviation and analyzed using independent samples *t*-tests. Non-normally distributed measurement data are presented as median (P25, P75) and analyzed using the rank-sum test. Categorical variables were expressed as frequencies and percentages, and compared using the chi-square test. Statistical significance was set at *P* < 0.05.

### Construction and validation of the prediction model

The target population was screened using the R language software based on inclusion and exclusion criteria. Missing values and outliers were assessed, non-numeric data standardized, and personal information removed. Significant factors from univariate and multivariate logistic regressions (*P* < 0.05) were used to construct a nomogram. Its clinical utility was then evaluated with decision curve analysis, and the factors were further used as inputs for machine learning models. Following feature selection via logistic regression, the dataset was partitioned into training and testing subsets using a 9:1 ratio within a 10-fold cross-validation framework. Multiple machine learning algorithms, including logistic regression, support vector machine, k-nearest neighbors, multi-layer perceptron, naive Bayes, decision tree, random forest, and gradient boosting, were trained and hyperparameter-optimized. The final model was selected based on predictive performance, with the dual objectives of mitigating overfitting and optimizing parameters. The use of multiple machine learning models allows for a comprehensive comparison of different algorithmic performances, helping to identify the most suitable approach for the specific dataset and thereby improving prediction reliability and robustness.

The receiver operating characteristic (ROC) curve is used to evaluate the overall performance of binary classification models.^11^ By varying classification thresholds, the dynamic relationship between the true positive rate (sensitivity) and the false positive rate (1 − specificity) is calculated, and the ROC curve is plotted to compute the area under the curve (AUC). By comparing the AUC values of different models, the model’s ability to distinguish between positive and negative classes is quantified, and the optimal threshold is determined based on the ROC curve to balance sensitivity and specificity.

The calibration curve is used to assess the reliability of the model’s predicted probabilities.^12^ By dividing the model’s output probabilities into several bins, the actual observed positivity rates within each interval are calculated and compared with the model’s predicted probabilities.

The net benefit curve is used to compare the performance of the logistic regression model with that of a theoretically perfect predictor, while also illustrating the net benefits associated with always taking action or never taking action strategies.^13^ The net benefit, commonly calculated using the formula net benefit = true positive rate − α × false positive rate, where α is a parameter that weighs the influence of false positive rate and true positive rate.

## Results

### Baseline data

This study included 401 patients who received HBOT after cerebral hemorrhage in our hospital. According to the mRS scores, patients were divided into a poor prognosis group (mRS < 3 points) and a good prognosis group (mRS ≥ 3 points). The baseline characteristics of the two groups of patients are compared in **Tables 1−3**. The poor prognosis group exhibited significantly higher initial CT hemorrhage volume, white blood cell count, prothrombin time, international normalized ratio, sodium concentration, blood glucose, time to HBOT initiation, intensive care unit stay duration, and total hospital stay duration compared to the good prognosis group. Conversely, the poor prognosis group exhibited lower GCS scores, ADL scores at both admission and discharge, and lower hemoglobin and albumin levels at admission.

**Table 1:** Basic characteristics of patients in the good and poor prognosis groups

	Good prognosis group (*n* = 265)	Poor prognosis group (*n* = 136)	*t*/*Z*-value	*P*-value
**Sex**				
Male	195(73.6)	98(72.1)	0.106	0.744
Female	70(26.4)	38(27.9)		
**Age (yr)**	58.51±14.37	59.2±12.86	–0.471	0.638
**GCS score on admission**	12.52±3.33	7.21±2.67	17.297	< 0.001
**ADL score on admission**	16.68±19.16	0.92±4.88	12.617	< 0.001
**CT hemorrhage volume (mL)**	16.21±9.93	34.27±14.53	–13.02	< 0.001
**Hypertension**	151(57)	82(60.3)	0.405	0.524
**Diabetes**	31(11.7)	22(16.2)	1.571	0.21
**Anticoagulant use**	39(14.7)	23(16.9)	0.331	0.565
**Hemiplegia**	130(49.1)	91(66.9)	11.582	0.001
**Aphasia**	34(12.8)	94(69.1)	131.033	< 0.001
**Etiology of cerebral hemorrhage**				
Spontaneous	161(60.8)	87(64)	0.394	0.53
Traumatic	104(39.2)	49(36)		
**Treatment modality**				
Conservative	180(67.9)	44(32.4)	48.166	< 0.001
Craniotomy	61(23.0)	74(54.4)		
Minimally invasive	24(9.1)	18(13.2)		
**Bleeding site**				
Basal ganglia	95(35.8)	60(44.1)	7.509	0.371
Thalamus	8(3)	3(2.2)		
Cerebral lobe	86(32.5)	35(25.7)		
Brainstem	2(0.8)	3(2.2)		
Cerebellum	9(3.4)	1(0.7)		
Subarachnoid hemorrhage	28(10.6)	17(12.5)		
Subdural space	26(9.8)	12(8.8)		
Epidural space	11(4.2)	5(3.7)		
IVH	33(12.5)	65(47.8)	60.789	< 0.001

Normally distributed measurement data are reported as mean ± standard deviation (SD) and analyzed using independent samples *t*-tests. Categorical variables were expressed as frequencies and percentages, and compared using the chi-square test. ADL: Activities of daily living; CT: computed tomography; GCS: Glasgow coma scale; IVH: intravascular hemolysis.

**Table 2:** Hyperbaric oxygen treatment and complications status of patients in the good and poor prognosis groups

	Good prognosis group (*n* = 265)	Poor prognosis group (*n* = 136)	*t*/*Z*-value	*P*-value
Initiation time of HBOT (d)	9.24±5.09	12.79±5.46	–6.441	< 0.001
Number of HBOT	8.55±3	8.46±3.74	0.265	0.791
Reasons for treatment interruption				
No interruption	182(68.7)	90(66.2)	14.13	0.004
Interruption due to complications	0	1(0.7)		
Patient intolerance	30(11.3)	29(21.3)		
Economic reasons	1(0.4)	0		
Oxygen toxicity	16(6)	22(16.2)	10.77	0.001
Barotrauma	15(5.7)	16(11.8)	4.695	0.03
Rebleeding	41(15.5)	50(36.8)	23.227	< 0.001
Infection	143(54)	129(94.9)	68.868	< 0.001
Thrombosis	91(34.3)	84(61.8)	27.484	< 0.001
Epilepsy	26(9.8)	55(40.4)	52.31	< 0.001
ADL score at discharge	37.74±24.06	3.49±6.16	16.318	< 0.001
Length of stay in the ICU (d)	3.06±5.53	10.18±6.99	–11.122	< 0.001
Length of hospital stay (d)	22.61±10.37	34.63±13.12	–10.01	< 0.001

Normally distributed measurement data are reported as mean ± standard deviation (SD) and analyzed using independent samples *t*-tests. Categorical variables were expressed as frequencies and percentages, and compared using the chi-square test. ADL: Activities of daily living; HBOT: hyperbaric oxygen therapy; ICU: intensive care unit.

**Table 3:** Laboratory indicators of patients in the good and poor prognosis groups

	Good prognosis group (*n* = 265)	Poor prognosis group (*n* = 136)	*t*-value	*P*-value
WBC (×10^9^/L)	10.52±5.03	11.56±4.59	–2.007	0.045
HB (g/L)	127.48±17.56	120.09±21.59	3.447	0.001
PLT (×10^9^/L)	185.53±73.57	176.04±63.5	1.279	0.202
PT (s)	13.74±1.25	14.39±1.98	–3.481	0.001
INR	1.07±0.11	1.14±0.21	–3.262	0.001
APTT (s)	35.54±13.56	34.05±4.78	1.245	0.214
D-dimer (ng/mL FEU)	6516.87±13811.75	7708.78±19441.99	–0.709	0.479
Total bilirubin (μM)	15.23±8.4	15.34±8.91	–0.125	0.901
ALT (IU/L)	24.92±27.89	32.85±62.24	–1.761	0.079
AST (IU/L)	28.79±21.14	50.78±166.59	–1.533	0.128
Albumin (g/L)	39.28±4.38	35.87±5.8	6.02	< 0.001
Potassium concentration (mM)	3.72±0.37	3.68±0.45	0.782	0.435
Sodium concentration (mM)	140.28±4.44	142.05±5.55	–3.218	0.001
Calcium concentration (mM)	2.17±0.18	2.18±0.95	–0.047	0.962
Serum creatinine (μM)	69.3±28.93	68.19±26.09	0.377	0.706
Uric acid (μM)	279.75±181.33	248.6±109.46	1.838	0.067
Blood glucose (mM)	7.58±2.48	8.32±2.36	–2.863	0.004

Normally distributed measurement data are reported as mean ± standard deviation (SD) and analyzed using independent samples *t*-tests. ALT: Alanine transaminase; APTT: activated partial thromboplastin time; AST: aspartate transaminase; FEU: fibrinogen equivalent units; HB: hemoglobin count; INR: international normalized ratio; PLT: platelet count; PT: prothrombin time; WBC: white blood cell count.

### Multivariate logistic regression analysis

The multivariate logistic regression analysis identified several independent risk factors associated with poor patient outcomes, including a lower admission GCS score (odds ratio (OR) = 0.65, 95% confidence interval (CI) 0.508 − 0.833, *P* = 0.001) and a higher admission CT hemorrhage volume (OR = 1.131, 95% CI 1.06 − 1.206, *P* < 0.001). Besides, a lower discharge ADL score (OR = 0.8, 95% CI 0.742 − 0.862, *P* < 0.001), non-surgical treatment (OR = 0.236, 95% CI 0.09 − 0.625, *P* = 0.004), presence of IVH (OR = 4.568, 95% CI 1.241 − 16.809, *P* = 0.022), and rebleeding (OR = 0.216, 95% CI 0.052 − 0.901, *P* = 0.035) were identified as independent risk factors (**Table 4**).

**Table 4:** Independent risk factors associated with poor outcomes in cerebral hemorrhage patients after hyperbaric oxygen therapy identified by multivariate logistic regression analysis

	*B*-value	OR	95% CI		*P* value
			Lower	Upper	
GCS score on admission	–0.43	0.65	0.508	0.833	0.001
ADL score on admission	–0.061	0.941	0.854	1.036	0.213
CT hemorrhage volume	0.123	1.131	1.06	1.206	< 0.001
WBC	–0.05	0.951	0.847	1.069	0.401
HB	0.006	1.006	0.971	1.042	0.74
PT	0.04	1.041	0.03	36.286	0.982
INR	2.555	12.873	0	15943618876964124.000	0.885
Albumin	0.005	1.005	0.876	1.154	0.939
Sodium concentration	–0.023	0.977	0.881	1.084	0.663
Blood glucose	0.076	1.079	0.898	1.296	0.417
Initiation time of HBOT	–0.028	0.973	0.85	1.113	0.686
ADL score at discharge	–0.223	0.8	0.742	0.862	< 0.001
Length of stay in the ICU	–0.026	0.974	0.86	1.103	0.679
Length of hospital stay	0.015	1.015	0.952	1.082	0.655
Treatment modality	–1.442	0.236	0.09	0.625	0.004
Hemiplegia	–0.568	0.567	0.16	2.011	0.38
Aphasia	0.473	1.604	0.431	5.967	0.481
IVH	1.519	4.568	1.241	16.809	0.022
Reasons for treatment interruption	0.473	1.606	0.959	2.688	0.072
Oxygen toxicity	0.446	1.562	0.134	18.186	0.722
Barotrauma	–1.433	0.239	0.021	2.688	0.246
Rebleeding	–1.533	0.216	0.052	0.901	0.035
Infection	0.46	1.584	0.3	8.372	0.588
Thrombosis	1.14	3.128	0.887	11.031	0.076
Epilepsy	0.514	1.672	0.442	6.328	0.449

ADL: Activities of daily living; CI: confidence interval; CT: computed tomography; GCS: Glasgow coma scale; HB: hemoglobin count; HBOT: hyperbaric oxygen therapy; ICU: intensive care unit; INR: international normalized ratio; IVH: intravascular hemolysis; OR: odds ratio; PT: prothrombin time; WBC: white blood cell count.

### Establishment and validation of the prediction model

Logistic regression analysis identified independent factors significantly associated with poor prognosis. The predictive ability of selected algorithms was evaluated using 10-fold cross-validation. The 10-fold cross-validation receiver operating characteristic curves showed the prediction results for various models: logistic regression (AUC = 0.98), support vector machines (AUC = 0.97), K-nearest neighbors (AUC = 0.96), multilayer perceptron (AUC = 0.97), naive Bayes (AUC = 0.97), decision tree (AUC = 0.96), random forest (AUC = 0.99), and gradient boosting (AUC = 0.98; **Figure 2**).

**Figure 2:**
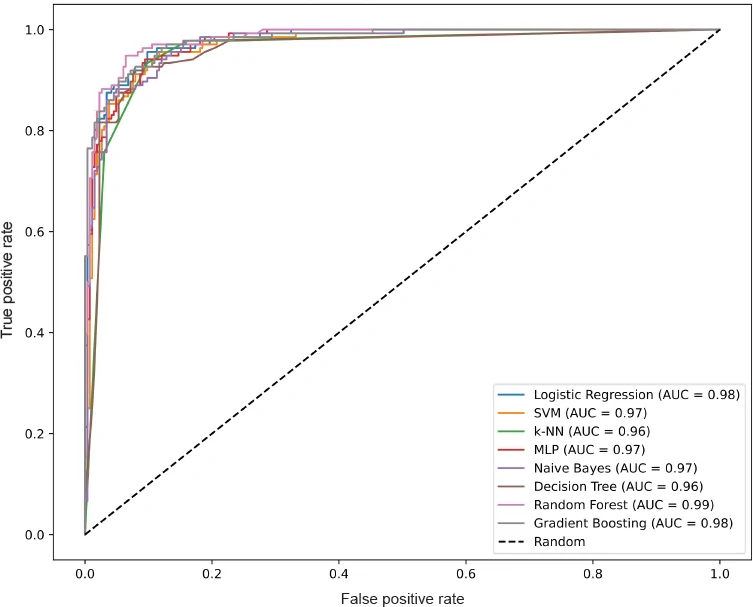
The comparison of ROC curves for eight different machine learning models for classification. The random forest model performed best (AUC = 0.99), followed by logistic regression and gradient boosting (AUC = 0.98). All models significantly outperformed the random guessing baseline (AUC = 0.5). AUC: Area under the curve; k-MN: k-nearest neighbors; MLP: multi-layer perceptron; ROC: receiver operating characteristic; SVM: support vector machine.

**Table 5** presents the predictive performance metrics for logistic regression and selected machine learning models, including average AUC, maximum AUC, and average ACC. Random Forest demonstrated superior predictive ability and accuracy. The calibration curve showed optimal alignment with the 45° reference line (**Figure 3**), indicating higher reliability. Net benefit curve analysis further supported the net clinical utility of the model (**Figure 4**).

**Figure 3:**
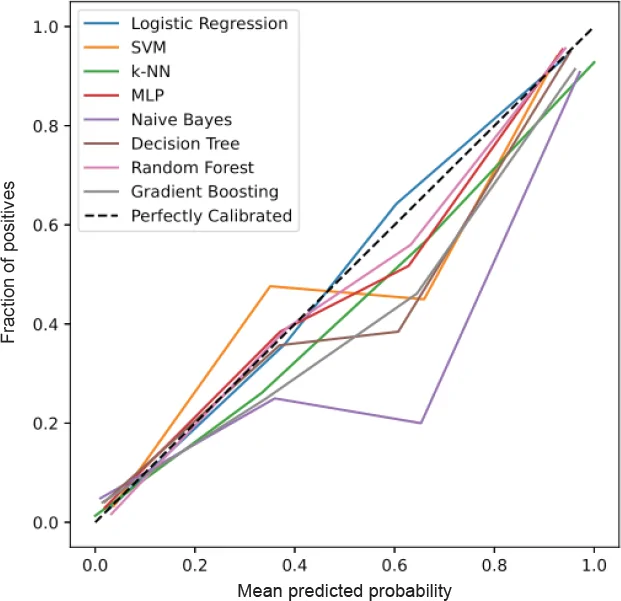
The reliability of the predicted probabilities of each machine learning model. The closer a curve is to the baseline (45° reference line), the better its calibration effect. k-MN: k-Nearest neighbors; MLP: multi-layer perceptron; SVM: support vector machine.

**Figure 4:**
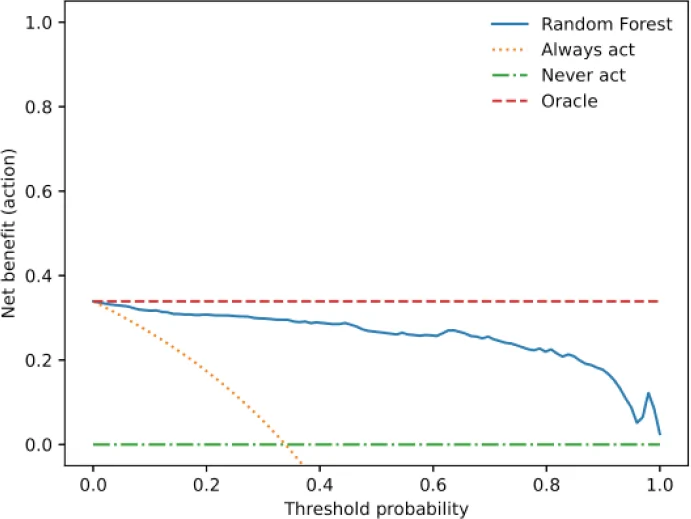
Decision curve analysis of the logistic regression model for prediction. The Oracle (red dashed line) indicates the achievable maximum net benefit corresponding to the theoretically perfect predictor; the net benefit of never act (green dotted line) is zero; always act (orange dashed line) indicates the net benefit obtained when always predicting positive.

**Table 5:** Predictive performance of eight machine learning models based on the test set

Model	Average AUC	Maximum AUC	Average accuracy
Logistic regression	0.98	1.00	0.93
SVM	0.97	0.99	0.91
k-NN	0.96	1.00	0.91
MLP	0.97	0.99	0.91
Naive Bayes	0.97	0.99	0.91
Decision tree	0.96	1.00	0.92
Random forest	0.99	1.00	0.93
Gradient boosting	0.98	0.99	0.92

AUC: Area under the curve; k-NN: k-nearest neighbors; MLP: multi-layer perceptron; SVM: support vector machine.

### Model interpretation of SHapley Additive exPlanations

To enhance the interpretability of the optimal random forest model, the SHAP method was employed. The SHAP bar chart in **Figure 5** depicts the relative importance of each feature, as measured by the average absolute SHAP value. The features are ranked in descending order of impact: discharge ADL score, CT hemorrhage volume at admission, GCS score at admission, IVH, treatment modality, and complication of rebleeding. The SHAP beeswarm plot in **Figure 6** visualizes the feature values and their corresponding SHAP values for each patient. The results were consistent with the feature importance ranking shown in the bar chart.

**Figure 5:**
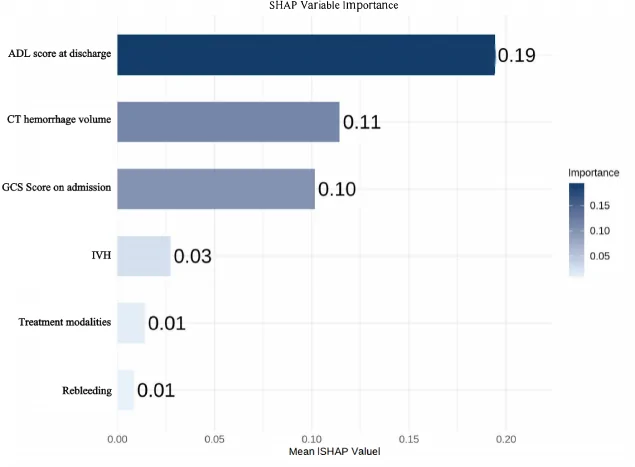
The interpretability of the optimal random forest model using SHAP method. The mean absolute SHAP values for each variable illustrate the ranking and relative importance of each variable in predicting poor prognosis. ADL: Activities of daily living; CT: computed tomography; GCS: Glasgow coma scale; IVH: intravascular hemolysis; SHAP: SHapley Additive exPlanations.

**Figure 6:**
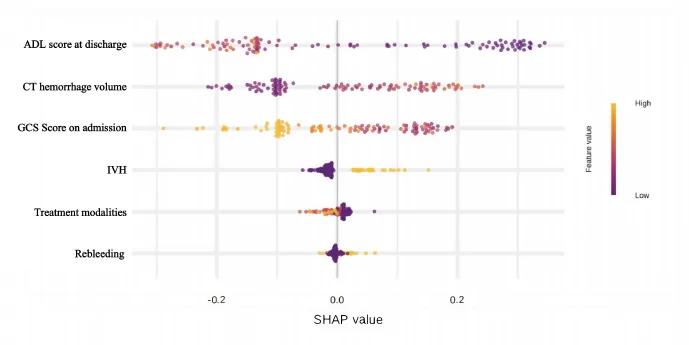
Beeswarm plot arranged based on the average absolute SHAP value, illustrating how variables influence the random forest model predictions. The positive SHAP value indicates a positive effect on the predicted outcome, and the negative SHAP value indicates an adverse effect. ADL: Activities of daily living; CT: computed tomography; GCS: Glasgow coma scale; IVH: intravascular hemolysis; SHAP: SHapley Additive exPlanations.

### Construction and validation of the nomogram

Finally, a nomogram was constructed based on the logistic regression model to visualize the contribution of each independent risk factor (**Figure 7**). The nomogram shows the values of each independent risk factor, determining the score by finding the corresponding points on each axis. Notably, the higher the CT hemorrhage volume at admission, the higher the values of IVH bleeding at admission and rebleeding, the higher the score. Conversely, the higher the values of surgical treatment, GCS score at admission, and ADL score at discharge, the lower the score. The higher the total score, the greater the risk of poor prognosis.

**Figure 7:**
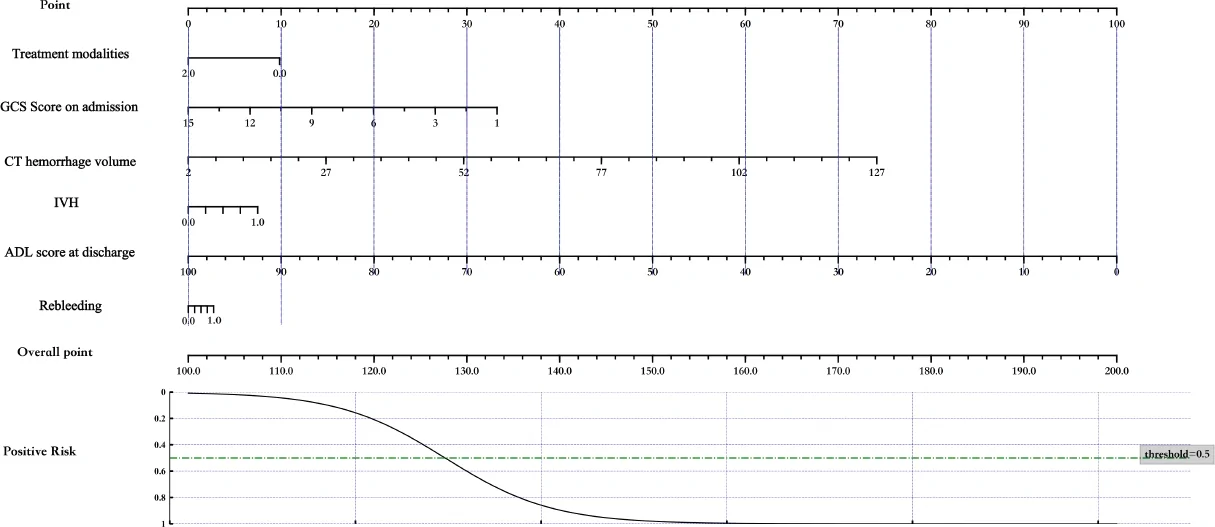
Prognostic nomogram for predicting poor prognosis risk after HBOT in patients with intracerebral hemorrhage. The higher the total score, the greater the risk of adverse outcomes. ADL: Activities of daily living; CT: computed tomography; GCS: Glasgow coma scale; HBOT: hyperbaric oxygen therapy; IVH: intravascular hemolysis.

## Discussion

Intracerebral hemorrhage is a critical neurological condition with high mortality and disability rates.^14^ HBOT has shown promise in improving patient outcomes. This study aimed to develop a predictive model for poor prognosis following HBOT in intracerebral hemorrhage patients. The random forest model outperformed others, consistent with previous studies on intracerebral hemorrhage prognosis.^15^,^16^ Ensemble methods, including gradient boosting and logistic regression, have demonstrated significant value in medical prognosis prediction.^17^,^18^ Despite its simplistic assumptions, naive Bayes yielded a comparable AUC to multilayer perceptron, possibly due to optimized feature engineering.^19^,^20^ Minor AUC variations across models suggest that high-quality clinical data can minimize the impact of different algorithmic structures on prediction performance.^21^ Traditional algorithms, including the support vector algorithm and K-nearest neighbors, maintained high AUC values, indicating that simple models can be as effective as complex ones in well-defined medical prediction tasks.^22^,^23^ Despite its slightly lower AUC, the decision tree model offers substantial value in clinical settings due to its interpretability. This study systematically developed and validated multiple machine learning models, providing a clinically interpretable predictive tool for the prognosis of intracerebral hemorrhage following HBOT, thereby addressing the gap in prognostic models for this specific treatment.

Analysis using the SHAP framework revealed that the Random Forest model achieved the best predictive performance. The model was well-calibrated, as evidenced by its close approximation to the 45°.^24^ Ensemble learning methods effectively integrate multidimensional features and enhance prediction stability for clinically heterogeneous data.^25^ SHAP value analysis identified key risk factors for poor prognosis after HBOT in cerebral hemorrhage patients: ADL score at discharge, CT hemorrhage volume at admission, GCS score at admission, IVH, treatment modality, and re-bleeding. ADL score at discharge, CT hemorrhage volume at admission, and GCS score at admission were the most influential predictors. ADL score, as a direct indicator of functional recovery, was the most important factor, emphasizing its critical role in long-term prognosis.^26^ Although the discharge ADL score is valuable for prognostic modeling, its assessment occurs following the completion of HBOT. Accordingly, this metric is unsuitable for initial risk assessment prior to treatment. Lower ADL scores are significantly linked to poor prognosis, indicating compromised functional recovery in patients, possibly due to irreversible nerve damage or complications. While HBOT can enhance neurological function in the acute phase, its long-term impact on daily living abilities may be limited, especially for patients with ventricular hemorrhage or re-bleeding. Higher ADL scores demonstrate a protective effect,^27^,^28^ guiding adjustments in HBOT treatment and suggesting the potential need for combined rehabilitation therapy. The initial CT hemorrhage volume and GCS score strongly predict injury severity. Large bleeding volumes correlate with positive SHAP values, reflecting hematoma expansion’s impact on neurological function.^29^ Hematomas exceeding 30 mL can lead to a mass effect, ischemic injury, and carry a high expansion risk.^30^,^31^ HBOT can improve the microenvironment but has limited reparative effects on large hematoma-induced structural damage. The interaction between hematoma volume and rebleeding risk requires dynamic imaging evaluation. A GCS score ≤ 8 indicates brainstem dysfunction or extensive injury, potentially limiting HBOT’s neuroprotective benefits.^32^,^33^,^34^

Consistent with these findings, the present study demonstrated that a decrease in GCS score was significantly associated with poor prognosis. Patients with lower GCS scores often exhibit a higher risk of intracranial hypertension and cerebral edema, which may offset the potential benefits of HBOT in improving cerebral oxygen metabolism.^35^ IVH is a key factor for poor prognosis in this study, as it may block cerebrospinal fluid circulation, exacerbate intracranial hypertension, and potentially induce an inflammatory response, leading to poor outcomes. Although HBOT can reduce cerebral edema, its ability to improve ventricular dilation or delayed hydrocephalus secondary to IVH is limited.^36^ The risk of poor prognosis is significantly increased by rebleeding events, as indicated by positive SHAP values, underscoring the importance of early prevention and treatment of rebleeding for improving outcomes. Rebleeding may be caused by poor blood pressure control or irregular shapes of intracranial hematomas. Since the anti-ischemic effect of HBOT may be offset by secondary damage after rebleeding, clinicians must carefully manage blood pressure and monitor coagulation function during the peri-treatment period. It is well-established that different treatment modalities have a substantial impact on prognosis. While surgical evacuation of hematomas may reduce the mass effect, the benefits of this approach combined with HBOT must be weighed against the risk of rebleeding. Conversely, patients undergoing conservative treatment may experience deterioration of neurological function due to delayed intervention. These differential effects suggest that individualized treatment strategies need to be comprehensively evaluated in combination with other patient characteristics.^37^,^38^

The logistic regression-based nomogram developed in this study quantitatively assesses the risk of poor prognosis following HBOT in cerebral hemorrhage patients by incorporating independent risk factors. As demonstrated in previous research,^39^,^40^ the nomogram translates key predictive factors, such as treatment modality, GCS score, CT bleeding volume, IVH, ADL score at discharge, and rebleeding, into objective risk probabilities via a visual scoring system. The positive correlation between the total score and the risk of poor prognosis aligns with prior studies on hypertensive intracerebral hemorrhage prognosis prediction.^39^ The calibration curve demonstrated a strong alignment with the 45° line of perfect agreement between predicted probabilities and actual outcomes, consistent with the brainstem hemorrhage model reported in the literature,^41^ confirming stable predictive accuracy in both training and validation sets. The proposed model demonstrated substantial net clinical utility through decision curve analysis, consistent with previous studies on intracerebral hemorrhage prognosis.^42^,^43^ This model may offer a more personalized predictive dimension by incorporating the HBOT responsiveness index, compared to the conventional intracerebral hemorrhage scoring system.^44^ However, we acknowledged that linear models may be inferior to machine learning algorithms, such as XGBoost, in capturing complex nonlinear relationships.^45^ Future improvements to the model’s predictive performance could be achieved by incorporating novel biomarkers, such as Radscore radiomics features^46^ or the platelet-to-white blood cell ratio.^47^ This study employed a single-center retrospective cohort design, which is inherently susceptible to information bias due to the reliance on the accuracy and completeness of electronic medical records. Although the cohort was stratified into two subsets, a training set and a testing set, the absence of multi-center external validation limits the generalizability of the results and highlights the necessity for future prospective studies to corroborate these findings. A significant limitation of the current research is the lack of comprehensive data related to essential HBOT treatment parameters, including the specific treatment pressure, duration per session, frequency of daily treatments, and individualized overall protocols. These parameters are widely recognized as crucial factors that may substantially affect both the biological response and clinical effectiveness of HBOT. Due to the retrospective data collection methodology, it was not feasible to consistently record or systematically analyze these parameters, thereby limiting their incorporation into predictive models and preventing comprehensive evaluation of their potential impact. To address these limitations, future prospective studies should emphasize the systematic and standardized acquisition of core HBOT treatment variables, thereby facilitating a more robust assessment of their influence on clinical outcomes and enhancing the interpretability of predictive analyses.

Therefore, this study integrates statistical and machine learning methods to identify crucial prognostic factors for patients with intracranial hemorrhage receiving HBOT. The random forest model shows strong predictive abilities, while SHAP analysis offers valuable insights into the relative importance of each risk factor. These findings are essential for prioritizing treatment, aiding personalized HBOT decisions, identifying potential beneficiaries, enhancing patient-doctor communication and rehabilitation goal setting, and dynamically monitoring disease progression’s impact on prognosis to adjust strategies promptly. Future research should aim to validate and expand the model’s applicability across diverse patient populations.

## Data Availability

*The raw data supporting the conclusions of this article will be made available by the authors, without undue reservation.*
